# Inflammatory Mechanisms in the Management and Treatment of Retinal Detachment

**DOI:** 10.3390/metabo15070442

**Published:** 2025-07-01

**Authors:** Pablo Redruello-Guerrero, María Gómez-Tomás, Tomás Rechi-Sierra, Laura Molinero-Sicilia, Nadia Galindo-Cabello, Ricardo Usategui-Martín, Salvador Pastor-Idoate

**Affiliations:** 1 Department of Ophthalmology, Hospital Clínico Universitario de Valladolid, 47003 Valladolid, Spain; 2 Unit of Excellence Institute of Applied Ophthalmobiology (IOBA), University of Valladolid, 47011 Valladolid, Spain; 3 Networks of Cooperative Research Oriented to Health Results (RICORS), National Institute of Health Carlos III (ISCIII), 28040 Madrid, Spain; 4 Department of Cell Biology, Genetics, Histology, and Pharmacology, Faculty of Medicine, University of Valladolid, 47003 Valladolid, Spain

**Keywords:** retinal detachment, neuroretinal inflammation, proliferative vitreoretinopathy, cystic macular edema, oxidative stress, retinal apoptosis, glia activation

## Abstract

Retinal detachment (RD) is a serious clinical condition that significantly impacts patients’ quality of life. Its management involves considering several clinical factors that may affect the therapeutic approach. Inflammatory complications can affect visual recovery, long-term outcomes, and prognosis. Understanding the underlying inflammatory mechanisms is key to improving personalized medicine and optimizing therapeutic approaches to management. This review comprehensively searched scientific databases (Medline, Web of Science, and Scopus), considering clinical and experimental studies published between 1999 and 2025. Specific MeSH terms and predefined inclusion and exclusion criteria were used to select the most relevant papers. A total of 140 studies were analyzed. The findings were analyzed qualitatively and illustrated with images from clinical practice. Several studies have demonstrated the critical role of cytokines in retinal inflammation, highlighting their importance in regulating the immune response following RD. In addition, oxidative stress, apoptotic mechanisms, and glia activation, particularly Müller cells and microglia, have been identified as crucial elements in the progression of retinal damage. In this sense, inflammation poses significant clinical challenges that require more effective therapeutic strategies. In conclusion, this review differs from previous literature by emphasizing the translational implications of inflammatory mechanisms in RD and by comparing experimental and clinical data. The management of RD should consider not only surgical aspects, but also modulation of the inflammatory response to improve visual outcomes and prevent long-term complications.

## 1. Introduction

Retinal detachment (RD) is caused by a separation of the photoreceptors (PRs) from the retinal pigment epithelium (RPE). As a specialized extension of the central nervous system (CNS), the retina relies on its laminar architecture, comprising neuronal and glial layers, to maintain visual processing and homeostasis. This neural tissue is housed within the immune-privileged environment of the eye, which includes the blood–retinal barrier and active immunosuppressive mechanisms that usually limit inflammation to preserve visual function. However, RD breaches both the anatomical integrity and immune balance of the retina, triggering inflammatory and oxidative responses that accelerate PR degeneration and compromise the delicate immune homeostasis of this CNS-derived tissue [1]. This breakdown of immune privilege facilitates the infiltration of immune cells and the release of pro-inflammatory mediators, initiating a cascade of neurodegenerative processes. Consequently, RD is considered an ophthalmologic emergency that can lead to severe vision loss if not treated promptly [2].

**Figure 1:**
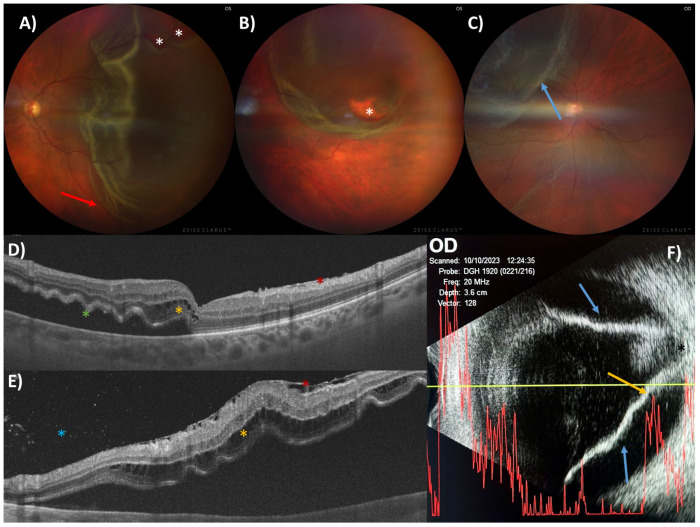
Alterations of retinal structures in patients with RRD. Color fundus photography (**A**–**C**), spectral-domain optical coherence tomography (SD-OCT) (**D**,**E**), and A- and B-mode ocular ultrasound (**F**) of several patients with RD. (**A**,**E**) correspond to the same patient. Fundus photography (**A**–**C**) enables visualization of retinal tears (indicated by the white asterisk) and the presence of retinal folds (red arrow), which may suggest that the lesion has been present for some time. (C) corresponds to a reRD showing a paler shade of the detached layers (indicated by the blue arrow). SD-OCT (**D**,**E**) shows multiple intraretinal cysts (yellow asterisk), epiretinal membrane (red asterisk), retinal neurosensory folds (green asterisk), Shaffer’s sign (blue asterisk), and subfoveal neuroretinal detachment. B-mode ultrasound (**F**) showing a funnel-shaped RD caused by a retinal detachment that respects the papillary zone (blue arrows). A-mode ultrasound shows a peak of hyperechogenicity corresponding to the detached neurosensory retina and a second peak of hyperechogenicity corresponding to the RPE (yellow arrow).

The most common type is rhegmatogenous RD (RRD), which happens when a tear or hole forms in the retina, allowing vitreous fluid to pass into the subretinal space [1,3]. The incidence of this pathology has been estimated at 13.3 cases per 100,000 population (95% CI: 11.3–15.6) [2,4]. However, the increase in incidence was 1.7 ± 0.1 cases per 100,000 person-years per year [5]. In this context, advances in surgical techniques have resulted in anatomical success rates exceeding 90%. However, visual outcomes are often limited due to permanent functional damage, especially when the macular area is affected (Figure 1) [6]. Since the macula is responsible for central vision, its involvement can severely impair tasks such as reading, driving, and recognizing faces—activities that are essential for independence and quality of life. Consequently, RD has a substantial impact on patients’ daily functioning and work performance, especially in individuals with lower socioeconomic status [7]. Furthermore, it is relevant to note that the economic cost of treating an RD ranges from USD 2763 to USD 7940, depending on whether pars plana vitrectomy (PPV), scleral cerclage, or pneumatic retinopexy is performed [8,9].

Despite advances in surgical techniques, visual recovery remains suboptimal in many cases, particularly due to persistent or unresolved inflammation. One of the key pathological events following RD is the disruption of the blood–retinal barrier, which impairs the exchange of metabolites between the neuroretina and the choroidal circulation. This disruption leads to ischemia and initiates neurodegenerative changes. Although the precise molecular mechanisms underlying retinal neurodegeneration after RD are not yet fully understood, apoptosis has been identified as a central contributor. Additionally, secondary factors like inflammation and oxidative stress exacerbate apoptosis, creating a neurotoxic environment that further damages the retina. Disruption of the blood–retinal barrier also results in increased chemotactic and mitogenic activity [10]. These factors are involved in the development of clinical complications, such as proliferative vitreoretinopathy (PVR), which remains the leading cause of failure after RD surgery [11]. PVR is also characterized by a strong inflammatory response, which plays a central role in its pathogenesis and progression.

Moreover, there is a lack of targeted pharmacological strategies aimed at modulating the immune response in the retina. These gaps underscore the need for a comprehensive review of the inflammatory pathways involved in RD, with the aim of identifying potential therapeutic targets to enhance long-term visual outcomes. This work aimed to review the published literature on the inflammatory pattern in the retina following RD, the role of inflammation in current treatments, and the long-term complications that may develop, and to evaluate the available therapies that act on the triggered inflammatory signals.

## 2. Materials and Methods

A comprehensive review of the main databases (Medline, Web of Science, and Scopus) was performed to collect the main research papers published between 1999 and 2025. The MeSH terms used were “retinal detachment”, “vitreoretinopathy proliferative”, “macular edema”, “neuroretinal inflammation”, “oxidative stress”, “retinal apoptosis”, “silicone oil”, and “glial activation”, which were combined using the Boolean operators “AND” and “OR”. In addition to MeSH terms, relevant synonyms and free-text keywords were used to capture studies that may not have been indexed under standard terminology. Filters were applied to include only original research articles and reviews published in English. Studies involving both human and animal models were considered, provided they addressed mechanisms of inflammation associated with RD or its complications. Articles written in languages other than English were excluded if full-text access or a reliable translation was not available.

## 3. Cell Death Mechanisms and Autophagy

The decrease in visual acuity in patients with macular involvement in RD is caused primarily by PR cell death. However, several interconnected and highly regulated molecular mechanisms underlie such degeneration.

### 3.1. Apoptosis and Necroptosis

PR death has been identified to begin 12 h after RD and to reach its peak at approximately 72 h. This has been widely confirmed in various animal models and human samples [12,13,14]. Several cell death pathways have been described following RD, including both apoptosis and necroptosis mechanisms. Apoptosis is a highly regulated process traditionally involving the activation of caspases. Among the primary pathways of PR death following RD is the extrinsic apoptosis pathway, triggered by the inflammatory cascade, which releases molecules such as tumor necrosis factor-alpha (TNF-α) and Fas ligand [15,16]. Inhibition of this pathway by the Met12 peptide targeting the Fas receptor prevents the generation of the caspase cascade induced by Fas ligand-Fas interaction. Besirli et al. [17] demonstrated that the outer nuclear layer (ONL) was significantly thicker in the treated group compared to mice not treated with this peptide. Simultaneously, the intrinsic pathway is activated through the release of specific pro-apoptotic molecules, such as Apaf-1, cytochrome C, or Smac/DIABLO, resulting in caspase-dependent mitochondrial damage [18].

Despite this, caspase inhibition has proven insufficient to prevent PR death entirely [13]. This limitation suggests the implication of caspase-independent pathways, such as one mediated by apoptosis-inducing factor (AIF). The migration of AIF from the inner segment of PRs to the cell nucleus supports this hypothesis [19]. Moreover, recent findings suggest that a type of necrosis may also be involved. Necroptosis has emerged as a programmed form of necrosis mediated by receptor-interacting protein (RIP) kinases. Unlike apoptosis, which is tightly controlled, necroptosis results in plasma membrane rupture and inflammation. This process appears to be especially stimulated when caspases are inhibited [20]. Consistent with this, elevated levels of RIP3 have been detected in RD retinas compared to nonpathological controls [21,22]. Moreover, the RIP1 kinase inhibitor notably reduced PR death, oxidative stress, and release of apoptotic factors [21].

These findings suggest that the interplay between apoptotic and necrosis pathways is key in PR degeneration. The extrinsic and intrinsic apoptotic pathways predominate in the early phases of the disease, primarily contributing to the initial PR cell death following RD. On the other hand, necroptosis arises as a compensatory mechanism, particularly when caspases are inhibited, initiating an uncontrolled inflammatory cascade with immune activation, microglial response, and damage to adjacent cells [23]. As a result, necroptosis may contribute to more aggressive photoreceptor (PR) degeneration, especially in the long term. Thus, simultaneous inhibition of both caspases and RIP kinases may offer a more effective neuroprotective approach for treating retinal neurodegeneration.

### 3.2. Autophagy

Autophagy is a complex catabolic mechanism that often plays a dual role, responsible for degrading and recycling intracellular components, including damaged proteins and organelles. This degradation occurs through the formation of autophagosomes that fuse with lysosomes, forming phagolysosomes, which have a strong degradative capacity [24]. The process is essential for maintaining cellular homeostasis, particularly under stress conditions such as oxidative damage or nutrient deficiency. In the ocular environment, autophagy removes damaged components and contributes to PR survival by mitigating cellular stress. Besirli et al. demonstrated that autophagy can negatively regulate caspase-dependent apoptosis, thus protecting retinal cells. However, autophagy’s protective role is context-dependent. When excessively activated, it can induce programmed cell death of cones in RD [25]. Different stress conditions can provoke this, including oxidative stress and hypoxia [26], both of which are main events occurring after RD. To protect themselves from such effects, retinal cells activate autophagy. However, when stress persists over time, sustained autophagic activation can lead to dysfunction due to lysosomal overload and a reduction in degradative capacity, as well as the degradation of critical components or proteins. Specifically, mitophagy has been observed as a mechanism to protect RPE cells from mitochondrial reactive oxygen species (ROS). However, when ROS levels are elevated, such as during age-related macular degeneration, RPE cell dysfunction has been associated with mitochondrial DNA damage [26].

On the other hand, there is an established relationship between autophagy and pyroptosis, as both processes can influence each other in the regulation of cell death mechanisms [27]. Pyroptosis is a form of programmed cell death characterized by an intense and acute pro-inflammatory nature. Unlike apoptosis, which is generally non-inflammatory and caspase-3-dependent, pyroptosis is mediated by caspase-1 via the inflammasome complex [28]. The balance between apoptosis and pyroptosis during the initial phase remains unclear and requires further investigation, as both appear to occur during the acute phase; however, the factors determining which pathway predominates are not yet established. Gasdermin D (GSDM) is a molecule involved in this process through its two subunits: GSDM-N, which mediates cell death, and GSDM-C, which inhibits it. Pyroptosis contributes to both local inflammation and the amplification of apoptotic and autophagic responses in the retina, thereby further exacerbating the initial neurodegeneration. Consistently, Li et al. [29] demonstrated that overexpression of GSDM-C can prevent GSDM cleavage, decreasing PRs’ cell death. These findings highlight the complex interplay between autophagy, inflammation, and cell death pathways.

## 4. Cytokines and Growth Factors in Regulating the Inflammatory Response After RD

**Figure 2:**
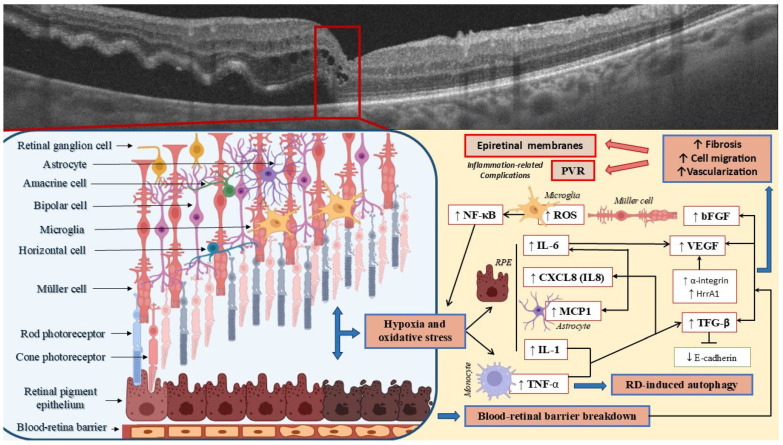
Schematic overview of the inflammatory and neurodegenerative cascade triggered by retinal detachment (RD). The top OCT image illustrates the separation between the retina and the underlying retinal pigment epithelium during retinal detachment (RD). The diagram below illustrates the retinal architecture and its cell types, highlighting the main events that occur after retinal damage (RD): breakdown of the blood–retinal barrier, hypoxia and oxidative stress, and the subsequent activation of inflammatory signaling pathways. Key cytokines (IL-6, IL-8/CXCL8, MCP-1, TNF-α, and IL-1) and growth factors (VEGF, TGF-β, and bFGF) are illustrated with their cellular sources and signaling effects, which can ultimately lead to chronic complications such as epiretinal membranes and PVR. The figure integrates the interplay between inflammation, autophagy, and neurodegeneration to highlight the complexity of RD pathology.

Cytokines are soluble proteins that act as key signaling molecules in the immune system. They regulate various biological processes, including immune cell activation, inflammation, and tissue repair [30]. The ocular immune response is primarily controlled by various interleukins (ILs), a cytokine group that modulates the activation, proliferation, and differentiation of immune cells involved in inflammation (Figure 2) [31]. Retinal cells also produce other types of cytokines, such as chemokines, which further contribute to immune regulation and cell signaling within the ocular system environment. In parallel, the retina secretes growth factors essential for maintaining retinal homeostasis, supporting cell survival, and reinforcing the eye’s immune privilege [32,33]. This privilege refers to a series of anatomical and molecular mechanisms that limit immune responses to prevent damage in tissues with limited regenerative capacity, thereby preserving visual function. These mechanisms include the blood–retinal barrier, lack of classic lymphatic drainage in the inner eye, immunosuppressive microenvironment, and active immune tolerance. However, under conditions of retinal injury or disease, cytokine production may be modified, often resulting in elevated levels of pro-inflammatory cytokines that disrupt the homeostatic balance [33]. A complex and robust inflammatory response is triggered following RD, further contributing to retinal damage and disease progression. Moreover, alterations in the retinal ganglion cell complex correlate with inflammatory biomarkers in the blood. Specifically, monocyte high-density lipoprotein showed a positive correlation with the mean thickness of the retinal ganglion cell complex (r = 0.511, *p* = 0.009) [34].

### 4.1. Interleukin-6 (IL-6)

IL-6 is a pleiotropic cytokine with both proinflammatory and anti-inflammatory actions. Classical signaling involves IL-6 binding to the receptor (IL-6R), which is mainly expressed in microglia. This pathway is generally anti-inflammatory, supporting functions such as oligodendrocyte differentiation, nerve regeneration, and PR survival. However, an alternative pathway is described, known as trans-singling, which involves the binding of IL-6 to a soluble form of IL-6R (sIL-6R). In this case, the pathway is pro-inflammatory, including the promotion of B cell differentiation, T cell proliferation, and NK cell activation [35].

In the retina, IL-6 is secreted by retinal pigment epithelium cells, T cells, and macrophages in the subretinal space as a response to inflammation [36]. In RD, higher levels were described in the vitreous group than in the control group [37,38,39,40,41,42]. Moreover, increased IL-6 has also been observed in the vitreous samples of patients who develop complications such as PVR and epiretinal membranes, both of which have a significant inflammatory component, compared to those who do not. This increase has been associated with the breakdown of the blood–retinal barrier [43,44]. The proangiogenic role of IL-6 is well-known in retinal pathologies [45]. In severe retinal detachment (RD) secondary to central retinal vein occlusion, increased levels of IL-6 and VEGF have been observed due to increased vascular permeability [46,47]. Similarly, upregulation of IL-6 has been observed in rat retinas following retinal ischemia/reperfusion injury. These data indicate activation of the alternative pathway involving sIL-6R, which triggers inflammation. In this scenario, modification of IL-6 levels has been tested as a regulator of it after RD. Intravitreal administration of exogenous IL-6 (150 ng) in albino rats maintained retinal ganglion cell density, compared to untreated animals [48].

Nevertheless, the anti-inflammatory effect of IL-6 has also been described in the context of retinal disease. As shown in experimental models, IL-6 can play a neuroprotective role for PRs [49]. Chong et al. demonstrated that IL-6^−/−^ mice had a higher percentage of apoptotic cells in the ONL than wild-type mice [49]. Similarly, neutralizing IL-6 with subretinal injection of an anti-IL-6 antibody increased the rate of apoptotic cells observed in Brown Norway rats, compared to the injection of vehicle or exogenous IL-6 [50]. Thus, exogenous addition of IL-6 may activate the classical pathway, contributing to PR survival by reducing apoptosis and limiting inflammatory damage. On the other hand, IL-6 regulates the expression of IL-10, a cytokine that has anti-inflammatory properties. However, studies show that IL-10 levels in the vitreous remain unchanged in patients with RD without postoperative complications and those with PVR [50,51].

### 4.2. C-X-C Motif Chemokine Ligand 8 (CXCL8)

CXCL8, also known as IL-8, belongs to the CXC chemokine family and has a potent proinflammatory function with chemotactic action for neutrophils and T lymphocytes [52]. Currently, it is known that CXCL8 can be secreted by RPE, activated microglia, Müller glia, and infiltrating immune cells such as macrophages and neutrophils [53]. In the eye, inflammatory factors stimulate its secretion primarily by the RPE due to the disruption of the symbiosis between the RPE and PRs [54]. Endothelial cells, monocytes, hypoxia, and oxidative stress affect the regulation of CXCL8 [55]. In retinal disease, mechanical stress exerted on the RPE immediately after RD stimulates the production of CXCL8, along with other signaling molecules, during the acute phase. Elevated levels of the chemokine have been observed in the vitreous of patients with RD, showing a 5.8-fold increase compared to healthy controls [42].

On the other hand, together with VEGF, CXCL8 promotes vascular permeability and ocular angiogenesis. This explains the elevated levels observed in vitreous hemorrhages after vitrectomy [56,57]. Additionally, the upregulation of CXCL8 and NF-kB, in both glial and vascular endothelial cells, appears to contribute to the formation of fibrocellular membranes, potentially favoring PVR pathogenesis [58]. Moreover, the expression of CXCL8 receptors, CXCR1 and CXCR2, in PVR retinas and membranes suggests a role for the chemokine in the activation and proliferation of these cells, and therefore, in the activation of gliosis [59]. Notably, a significant correlation between IL-6 and CXCL8 (r = 0.645; *p* < 0.0001) has also been described [60] in patients with PVR.

### 4.3. Monocyte Chemoattractant Protein (MCP-1)

MCP-1, or CCL2, is produced by RPE cells and astrocytes after RD, exerting an indirect cytotoxic effect on PRs. Its levels are elevated in Müller glial cells, which increases the presence of macrophages and microglia activation. The increase in MCP-1 leads to heightened oxidative stress activation, which amplifies PR apoptosis, a form of programmed cell death, and the primary cause of vision loss after RD. However, an in vitro study showed that selective removal of macrophages and microglia in cell cultures did not affect PR survival [61]. IL-1 and TNF-α stimulate MCP-1 in fibroblasts, endothelial, epithelial, and RPE cells [62]. Interestingly, while IL-8 and MCP-1 appear to be involved in the initial events of RD [63], their levels can be elevated in the later stages as well. This occurs because phagocytes and lymphocytes begin to release more IL-1 and TNF-α, which further stimulate the secretion of IL-8 and MCP-1, along with other chemoattractants for immune cells, such as transforming growth factor-beta (TGF-β). Thus, the inflammation process is amplified, and fibroproliferation is favored, potentially leading to visual complications [64].

### 4.4. Tumor Necrosis Factor Alpha (TNF-α)

TNF-α is primarily secreted by monocytes, and its effects have been associated with neuronal degeneration. Its expression after RD shows a biphasic pattern, with peaks at 1 and 6 h, indicating an active involvement in the acute inflammatory phase. Although it may not play a crucial role in acute RD without complications, compared with other cytokines such as IL-6, elevated levels of TNF-α appear to contribute to the long-term degeneration of PRs [65]. Specifically, TNF-α induces apoptosis of PRs through activation of the TNF-α type 2 receptor, and increases its expression in patients with proliferative disorders, such as PVR. This makes it a potential biomarker for postsurgical complications. In this context, a study reported that the rs2229094 polymorphism in the *LTA* gene in the *TNF* gene may increase the risk of suffering PVR [66]. Supporting these findings, elevated mRNA levels of *TNF-α* and its receptors have been detected in the neuroretina of pigs under ischemic conditions [67]. Anti-TNF-α therapies have demonstrated in vitro efficacy in promoting the survival of PRs [15]. Moreover, a study indicates a significant part of TNF-α in RD-induced autophagy, and suggests that modulating this pathway could offer a therapeutic strategy to protect PRs [16]. Infliximab, a monoclonal antibody that suppresses TNF-α, has demonstrated attenuation of autophagy in murine models by decreasing the expression of LC3B and Atg5 proteins [16]. Overall, TNF-α appears to act as a mediator in both the early inflammatory response after RD and, more importantly, in the progression towards chronic complications.

### 4.5. Growth Factors

Growth factors are small glycoproteins that regulate cell communication [68]. They play a crucial role in modulating inflammatory responses within the retina (Figure 2). More specifically, they enable continuous interaction between nearby and distant cells, modulating cellular behavior and helping maintain tissue structure and function. They are key in normal physiology, involved in processes like tissue remodeling and angiogenesis, as well as the activation of immune cells [68]. On the other hand, their significant roles in pathological conditions like diabetic retinopathy have been widely described [69,70]. Notably, the use of these molecules for therapeutic reasons is growing [71,72,73,74,75,76,77,78].

#### 4.5.1. Vascular Endothelial Growth Factor (VEGF)

VEGF is the most recognized growth factor, playing a crucial role in proliferation, inflammation, and angiogenesis [79]. In the ocular context, increased vitreous VEGF levels in primary RD have been noted due to inflammation and oxidative stress, retinal hypoxia, and breakdown of the blood–retinal barrier. [56,80]. Moreover, damage caused by *VEGF* expression can lead to scarring and fibrosis, further compromising visual function. Thus, high VEGF levels are often associated with a bad prognosis in retinal disease [81]. Moreover, α5β1 integrin has been shown to participate in VEGF-mediated vascular proliferation in RD [82]. This integrin promotes endothelial cell adhesion and migration, processes enhanced by VEGF signaling. The effect of ATN-161, an inhibitor of this integrin, demonstrated a lower rate of RD when induced by aberrant vessel proliferation driven by VEGF [72]. High-temperature requirement factor A1 (HtrA1) has also been associated with VEGF expression due to cellular stress caused by RD [83]. Therapeutic interventions aimed at reducing VEGF levels can help mitigate these harmful effects and protect retinal tissue from excessive scarring and fibrosis that ultimately compromise the functional integrity of the eye. Intraocular statin treatment has shown lower VEGF levels in patients with RD and a gain in visual acuity [73]. Additionally, simvastatin, through its modulation of inflammation, including the reduction of VEGF, has demonstrated preventive effects against the development of inflammatory complications, such as PVR and re-RD [74] Similarly, Zhao et al. [75] showed that administration of IL-10, an interleukin that negatively regulates VEGF, decreased the expression of the growth factor in RPE.

Importantly, basal levels of VEGF are crucial for retinal homeostasis. Under physiological conditions, VEGF supports endothelial cell survival, promotes vascular repair, and helps limit inflammation. In experimental models of retinal ischemia, VEGF-A and anti-inflammatory factors are produced following Par-2 activation in retinal neurons. This activation is triggered by expression of proteinase-activated receptor (Par-2), which is upregulated in response to IL-1β release, that initiates a cascade that culminates in endothelial apoptosis [76]. In this context, VEGF expression plays a role in limiting this apoptosis. Thus, while excessive VEGF activity can be detrimental, complete inhibition of this growth factor may be counterproductive.

#### 4.5.2. Basic Fibroblast Growth Factor (bFGF)

bFGF, also known as FGF2, is a member of the fibroblast growth factor (FGF) superfamily and another key factor in initiating angiogenesis [77]. It can also enhance leukocyte recruitment to inflamed sites [78] and has shown a neuroprotective effect on retinal tissue by preventing PR degeneration after RD. Such neuroprotection has been hypothesized to come from either indirect activation of Müller cells or increased PR expression [55]. On the other hand, bFGF appears to be a key molecular mediator in the pathogenesis of PVR, as its presence in the vitreous cavity is directly linked to the disruption of the blood–retinal barrier, significantly contributing to the proliferative and contractile processes of this complication [10]. Consequently, its levels are found to be elevated in PVR membranes and vitreous [84]. One of the most significant mechanisms by which bFGF contributes to this pathology is by indirectly activating the platelet-derived growth factor receptor alpha (PDGFR-α). Both VEGF and bFGF can act synergistically, activating similar signaling pathways, such as the one involving PDGFR-α [10]. bFGF has also been shown to stimulate both RPE and glial cells. Müller cells are a major source of this factor in the ischemic retina, as prolonged hypoxia triggers its release. This, along with VEGF, promotes the proliferation of retinal endothelial cells and can contribute to pathological neovascularization [84].

#### 4.5.3. Transforming Growth Factor-β (TGF-β)

TGF-β is a multifunctional cytokine that regulates both immune responses and tissue remodeling. It contributes to the eye’s immune privilege by inhibiting antigen-driven T cell activation and proliferation. It also promotes epithelial–mesenchymal transition (EMT) and extracellular matrix (ECM) production in RPE cells, facilitating their migration into the vitreous cavity [85]. The upregulation of ECM is often accompanied by a downregulation of E-cadherin, promoting the loss of cell adhesion and increasing tissue invasiveness [86]. Additionally, TGF-β-induced fibroblast activation stimulates collagen synthesis, contributing to the stiffness of epiretinal membranes [87]. Hinton et al. [70] established that TGF-β activation also upregulates the expression of connective tissue growth factor (CTGF). This signaling interplay drives RPE cells to adopt a myofibroblast-like phenotype, thereby initiating fibrotic processes that generate tractional forces on the retina, promoting further detachment and perpetuating retinal damage. Consistently, levels of TGF-β are found to be increased in patients with PVR. However, a study indicates that only PVR-A (not PVR-B nor PVR-AC) showed a statistically significant increase correlated with the duration of RD. This suggests a role of TGF-β primarily in the initial part of the process [88].

## 5. Oxidative Stress in RD

Inflammatory responses result in activation of infiltrating microglia and macrophages, leading to the release of proinflammatory cytokines such as IL-1β and TNF-α. These inflammatory mediators stimulate the production of reactive oxygen species (ROS), contributing to oxidative stress. In turn, oxidative stress activates redox-sensitive signaling pathways such as NF-κB, further amplifying the inflammatory response. This mutual activation creates a self-perpetuating cycle that accelerates PR degeneration and disrupts the blood–retinal barrier. In this context, modulating the oxidative-inflammatory axis becomes critical for therapeutic intervention. Adenosine, a neuromodulator in the central nervous system, plays a key role in regulating oxidative stress and promoting neuroprotection in the retina by activating four G-protein-coupled receptors: A1, A2A, A2B, and A3. A2A receptors have been identified in retinal microglia, where they modulate inflammation and oxidative stress. Gao et al. [89] found that A2A expression is increased in microglia and Müller cells of the detached retina in mice, leading to heightened reactivity in these two cell types. Moreover, selective antagonism of the A2A receptor by ZM241385 effectively protected PR by suppressing microglia activation, proinflammatory IL-1β cytokine production, and ROS generation. ZM241385 also inhibited the upregulation of glial fibrillary acidic protein (GFAP) expression in Müller cells.

NADPH oxidase (NOX) activation affects PR degeneration in a directly proportional manner [90]. Adrenoceptor ligands, such as α1 antagonist (doxazosin) and α2 agonist (guanabenz), protected PRs from apoptosis and preserved retinal function. These compounds acted by inhibiting the overproduction of ROS, reducing the expression of inflammatory cytokines such as IL-1β and CCL2, and suppressing gliosis. Therefore, treatment with doxazosin and guanabenz showed neuroprotective effects. However, the use of guanabenz at high doses caused side effects such as drowsiness, hypotension, and sedation, which are dose-dependent and related to its central α2-adrenergic activity. Further studies are needed to define its therapeutic window and long-term safety [90].

Antioxidants play a crucial role in regulating ROS production. One such antioxidant, edaravone (3-methyl-1-phenyl-2-pyrazolin-5-one), a potent scavenger of hydroxyl radicals, could be a potential neuroprotectant [91] in PR. Excess ROS generation leads to oxidative modifications of essential biomolecules, including DNA, lipids, and proteins, triggering cellular dysfunction and apoptosis. Lipid peroxidation, in particular, is a key pathological feature in RD and is commonly assessed by the accumulation of 4-hydroxynonenal (4-HNE), a toxic byproduct. In murine models of RD, 4-HNE levels were significantly elevated in the inner plexiform layer (IPL), inner nuclear layer (INL), and outer plexiform layer (OPL) of the detached retina compared to regions where the retina remained adherent. Treatment with edaravone effectively reduced 4-HNE accumulation in these retinal layers, suggesting a mitigation of lipid peroxidation. Moreover, edaravone also decreased caspase activation and the number of apoptotic cells, indicating its broader anti-apoptotic and neuroprotective potential [91]. Although edaravone may be a promising treatment to control ROS production and PR apoptosis, the optimal dosage should be evaluated in future studies. Notably, edaravone is already approved for clinical use in amyotrophic lateral sclerosis, which supports its translational potential as a neuroprotective agent [92].

Green tea extract (GTE) is an antioxidant, anti-inflammatory, and anti-apoptotic agent that has been studied as a bridging treatment to control inflammation until surgery [93]. For this purpose, a rat model with induced RD was used, and GTE was administered orally for three days. A low dose of GTE exerted protective effects, evidenced by reductions in retinal edema after RD confirmed by increased a-and b-wave amplitudes in the electroretinogram, decreased ONL thickening, and significant reduction of apoptotic cells [93]. GTE suppresses the accumulation of hypoxia-inducible factor 1α (HIF-1α) and exerts an anti-inflammatory effect by inhibiting the production of inflammatory cytokines, such as CCL2 [94], while increasing anti-inflammatory cytokines, such as IL-13 [93]. This evidence suggests that the antioxidant and anti-inflammatory effects of GTE are not merely additive but may act synergistically. In a rat model of RD, low-dose GTE significantly reduced oxidative stress and inflammation through interconnected pathways, including suppression of HIF-1α and modulation of cytokine profiles.

Forkhead box O (FoxO) transcription factors are a family of proteins that regulate gene expression to modulate various cellular processes, including the cell cycle, apoptosis, DNA repair, stress resistance, and cellular metabolism. Activated FoxO proteins cause a decrease in oxidative stress by binding to the promoters of genes encoding specific scavenger proteins that play an essential role in oxidative detoxification [95]. Resveratrol, a polyphenolic flavonoid with potent antioxidant activity, increased the expression of the FoxO1A, FoxO3A, and FoxO4 transcription factors. Intraperitoneal injection of 20 mg/kg in Brown Norway rats with induced RD demonstrated reduced apoptosis of PRs and inhibited caspases 3, 8, and 9 [95].

## 6. Chronic Inflammation

Chronic inflammation in RD may play a key role in the progression of ocular damage and in postsurgical evolution. It induces epithelial and glial cell proliferation, favoring the formation of epiretinal membranes and the development of PVR [11]. One of the most detrimental effects of chronic inflammation is the development of epiretinal fibrosis and the proliferation of preretinal and subretinal membranes. These structural changes can induce retinal traction and increase the risk of recurrent RD. The transition from macrophages to myofibroblasts appears to play a prominent role in the development of epiretinal membranes in PVR. Specifically, this transition seems to involve macrophages with a predominant M2 phenotype, which acquire mesenchymal characteristics, including the expression of α-smooth muscle actin (α-SMA). The macrophage-to-myofibroblast transition includes the involvement of TGF-β1/Smad signaling and other fibrogenic pathways [11]. A research group [96] found that CD68+ macrophages in epiretinal membranes express myofibroblast markers, indicating that this cell transition contributes to the fibrosis characteristic of PVR. Furthermore, the elevated levels of soluble fibroblast activation protein alpha (sFAP-α) in the vitreous suggest that it may serve as a biomarker of macrophage activation in this context. According to the study, sFAP-α may be derived from both M2 macrophages that transition into myofibroblasts and Müller cells. However, its clinical utility as a predictive biomarker for PVR remains experimental. Although increased sFAP-α levels have been associated with fibrotic activity, no validated thresholds, sensitivity, or specificity values have yet been established for clinical use. Further studies are needed to determine its prognostic value and potential for early detection of PVR.

As previously mentioned, TGF-β plays a fundamental role in the pathophysiology of PVR. This factor is mainly stored in PRs, although choroids have also been described [97]. In the presence of TGF-β2, hyalocytes transdifferentiate into myofibroblasts and may favor the contraction of retinal fibrosis [98]. In human RPE cells, TGF-β2 activates multiple signaling pathways that contribute to epithelial–mesenchymal transition (EMT) and extracellular matrix (ECM) production. These include the canonical Smad2/3 pathway, as well as non-canonical pathways such as the PI3K/Akt and p38 MAPK pathways. Activation of these cascades leads to downregulation of epithelial markers (e.g., E-cadherin) and upregulation of mesenchymal markers (e.g., α-SMA and fibronectin), promoting cell migration, contractility, and fibrotic remodeling—hallmarks of PVR progression [99].

On the other hand, MCP-1 levels in the vitreous fluid correlate with IL-6 levels and the severity of PVR [64]. An in vitro model with human RPE cells demonstrated that MCP-1 stimulates RPE cell migration in a dose-dependent manner, and that dexamethasone inhibits such migration [100]. Importantly, these findings have been corroborated in animal models, where MCP-1 overexpression has been linked to increased subretinal fibrosis and PVR-like changes. Moreover, clinical studies have reported elevated MCP-1 levels in the vitreous of patients with PVR, supporting its translational relevance as a potential therapeutic target [64]. In addition, bFGF increases the proliferation of Müller cells, retinal astrocytes, RPE cells, and fibroblasts in vivo [43] and is elevated in the vitreous of eyes developing PVR [101]. Recently, it has been reported that Müller glial cells play an essential role as a source of proinflammatory cytokines and growth factors involved in PVR-associated retinal gliosis [91]. Both α1-adrenergic receptor antagonist and α2-adrenergic receptor agonist could protect PRs against apoptosis and reduce Müller glial cell activation. Therefore, it is still necessary to investigate whether α-adrenergic receptor ligands could effectively prevent PVR progression after RD.

On the other hand, Lumi et al. [102] suggested that certain genetic variations in the *SOD2* and *IL1B* genes may be associated with the risk of developing PVR after RD. In particular, the *IL1B* rs1071676 polymorphism seems to be associated with better postoperative visual acuity in patients without PVR, suggesting that specific genetic profiles may predispose or protect against this postsurgical complication. However, these findings are still preliminary, and broader validation in larger, diverse cohorts is needed before considering routine genetic screening for PVR risk stratification. Inflammatory cytokines and growth factors, such as PDGF-AA, TGF-α, VEGF, IL-6, IL-8, and TNFβ, are significantly elevated in patients with PVR. However, other authors [103] have demonstrated that inflammation is predominantly localized to the retina, as circulating levels of these biomarkers were not detected in the blood.

## 7. Glial Activation: Müller Cells and Microglia

In the retina, several types of glial cells play essential roles in maintaining homeostasis, protecting neurons, and responding to injury. The central retinal glia are Müller cells, microglia, and astrocytes. Müller cells are the most abundant type of glia and extend radially through almost all retinal layers, providing structural support, regulating ionic balance, recycling neurotransmitters, and offering antioxidant defense. Microglia, the resident macrophages of the central nervous system, remain in a state of immune surveillance and are rapidly activated in pathological contexts. Astrocytes, located mainly in the nerve fiber layer, participate in the formation of the inner blood–retinal barrier and the metabolic support of the optic nerve. The interaction between these glial cells is key in both physiological conditions and retinal diseases [104].

Various chemokines, including TNF-α, IL-1β, IL-6, IL-8, and MCP-1, can reach elevated levels within an hour of RD. These chemokines contribute to microglial activation and the recruitment of macrophages into the subretinal space. Once activated, microglia can release MCP-1, further enhancing its expression in Müller cells. This feedback loop amplifies the inflammatory response in the retina, potentially harming PRs [1,55].

**Figure 3:**
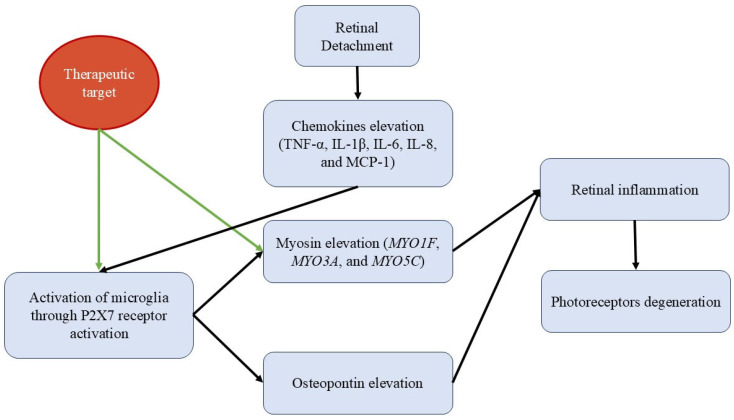
Scheme of microglia activation. The mediators involved in retinal inflammation following RD and the primary therapeutic targets under investigation are illustrated in the figure.

The myosin family, a crucial component of the cytoskeleton, has been reported to play a significant role in cell signaling. It can regulate the MAPK and AKT pathways to promote the transcription of proinflammatory cytokines and activate microglia. The MAPK and ATK pathways are known to be crucial for the release of proinflammatory cytokines from microglia. Wang et al. [105] investigated the involvement of myosin 1F (*MYO1F*) in microglia activation through regulation of MAPK and AKT signaling pathways in a murine model of PR degeneration secondary to RD. A total of 38 human retinal samples were analyzed, comprising 19 from patients with RD and 19 from controls without it. Gene expression analysis revealed a significant increase in *MYO1F*, *MYO3A*, and *MYO5C* levels in the RD samples, with *MYO1F* exhibiting the highest growth among the myosins. To assess the temporal dynamics of *MYO1F* expression after RD, patients were categorized into three groups according to the duration of RD (<1 month, 1–3 months, and >3 months). The highest *MYO1F* levels were observed in the group with RD of less than 1 month of evolution. Immunofluorescence confirmed an increase in microglial activation markers associated with *MYO1F* overexpression. To assess the functional role of *MYO1F* in PRs degeneration, a *MYO1F*-deficient mouse model was used, with typical structures observed in both optical coherence tomography and histological sections with hematoxylin-eosin, suggesting that the absence of *MYO1F* confers protection against PRs apoptosis. These findings indicate that the increase in *MYO1F* leads to greater microglial activation and, consequently, higher expression of inflammatory cytokines, thereby increasing PRs damage. Consequently, *MYO1F* may represent a promising therapeutic target for preserving retinal function and preventing vision loss in retinal degenerative diseases (Figure 3).

It has been established that P2X7 receptors are located along the membrane of microglia. Activation of P2X7 by ATP promotes the release of proinflammatory cytokines, particularly IL-1, which in turn induces pyroptosis. A recent study conducted in China in 2023 [106] investigated, using murine models, the role of ATP-stimulated P2X7 receptor activation in the migration and activation of microglia into the subretinal space. The study found that acute separation of PRs and RPE generates ATP accumulation in the subretinal space, which promotes microglia recruitment through P2X7 activation and triggers PR pyroptosis. Furthermore, pharmacological blockade of P2X7 effectively prevented microglial migration and reduced PR death, both in the acute phase of RD and in the long term. In this context, downregulation of P2X7 by Brilliant Blue G, a specific inhibitor of this receptor, showed effective inhibition of microglial migration in vivo and attenuation of the inflammatory response of microglia in vitro. Although Brilliant Blue G has been safely used in humans as an ophthalmic dye during surgeries, its use as a P2X7 inhibitor has so far been limited to preclinical studies. No clinical trials have been reported to date evaluating this application in humans.

Osteopontin (OPN) serves as a marker for microglial activation linked to heightened retinal inflammation and is primarily localized near the RD [107]. Iba-1-positive microglial cells were identified in the subretinal space of the detached and borderline regions between days 3 and 14 post-RD, reaching a maximum peak of expression at day 7, with no presence in the intact regions. This study also aimed to validate OPN as a specific marker of retinal inflammation after RD-induced injury. It was confirmed that OPN is positively regulated and secreted by activated microglial cells and that its expression was observed exclusively in affected regions, not in intact areas. The correlation between OPN expression and cell death in retinal injury has been previously reported in RD and glaucoma, supporting its usefulness as a biomarker of retinal inflammation associated with tissue damage [107]. Even so, the study does not provide direct evidence of a correlation between OPN expression and clinical severity or outcomes in patients with RD. The research is limited to an animal model, and no clinical studies are mentioned that validate OPN as a biomarker in humans samples.

## 8. Genetic Modulation of Inflammatory and Apoptotic Responses: Implications for Personalized Therapies After RD

Evidence shows that individual genetic backgrounds modulate the response to RD, particularly by influencing inflammation, oxidative stress, and programmed cell death mechanisms [12,102,108,109,110]. Genetic polymorphisms account for the variability in clinical outcomes and the risk of postoperative complications, including PVR.

### 8.1. Genetic Variants Associated with Apoptosis, Inflammation, and Oxidative Stress Genes: Clinical Examples

Apoptosis plays a central role in PR degeneration following RD. Genetic variations in key apoptotic regulators, such as BAX and BCL-2, have been associated with differential susceptibility to PVR. The *BAX-248G > A* polymorphism has been linked to a higher risk of developing PVR, particularly in individuals homozygous for the A allele, which is associated with reduced pro-apoptotic activity. In a Spanish cohort, carriers of the AA genotype had a 1.8-fold increased risk of PVR compared to those with the GG genotype. Conversely, the *BCL-2-938C > A* variant appears to exert a protective effect, especially in southern European populations, where the AA genotype (linked to increased Bcl-2 expression and anti-apoptotic activity) was associated with a 50% reduction in PVR risk [111]. Similarly, the tumor suppressor Tp53 exhibits genetic variability that influences RD outcomes. The codon 72 polymorphism (rs1042522) affects apoptotic capacity, with homozygosity for the Proline variant significantly increasing the risk of PVR [112]. Further modulation occurs through *MDM2*, where the T309G polymorphism promotes *MDM2* overexpression, leading to greater degradation of p53 and reduced apoptotic clearance. This correlation is associated with higher PVR susceptibility [113].

Beyond apoptosis, oxidative stress and inflammatory responses are critically involved in RD pathophysiology [102,109]. Polymorphisms, such as rs4880 in *SOD2* and rs10716 in IL1B, have been associated with an increased risk of PVR [102]. *SOD2* encodes a mitochondrial antioxidant enzyme essential for neutralizing reactive oxygen species, while IL1B regulates key inflammatory pathways. Alterations in these genes may exacerbate retinal injury by amplifying oxidative damage and inflammatory cascades [102,109]. Although these genetic associations offer valuable insight into the molecular mechanisms behind PVR susceptibility, their application in clinical practice remains limited. Currently, none of these variants are routinely used for patient stratification or therapeutic guidance. Larger, multiethnic validation studies are necessary to establish their predictive value, define risk thresholds, and evaluate cost-effectiveness before genetic screening can be incorporated into standard clinical workflows.

### 8.2. Complex Genetic Architecture: Interplay Between Genetic and Environmental Factors

Although most cases of RD result from acquired factors, genetic background can modulate individual susceptibility. Specific mutations in structural genes, such as *COL2A1* and *COL11A1*, particularly in conditions like Stickler syndrome, compromise vitreoretinal architecture and significantly increase susceptibility to RD [12,108]. Additionally, genome-wide association studies (GWAS) have suggested that up to 27% of the heritability of primary RD may be attributable to common variants identified so far, highlighting its multifactorial nature [12,108]. Notably, GWAS have identified loci near genes such as *SS18*, *PSAM8*, *TSTA3,* and *LDB2*, which are involved in extracellular matrix remodeling, cell adhesion, and retinal development [114]. These findings could inform future risk prediction models by integrating polygenic risk scores to stratify individuals based on genetic susceptibility. Moreover, identifying at-risk individuals through genetic profiling may inform personalized monitoring strategies or inform the inclusion of individuals in preventive clinical trials.

These findings underscore that while genetic predisposition plays a critical role, it interacts closely with environmental factors. Understanding this interplay is vital for a comprehensive view of RD pathogenesis and for advancing personalized therapeutic strategies.

### 8.3. Implications for Clinical Management

Building upon the genetic framework, external factors such as vitreous liquefaction, ocular trauma, and surgical intervention critically influence the onset and severity of RD and its complications [110]. A genetically primed pro-inflammatory or anti-apoptotic environment may exacerbate tissue damage, promote fibrotic remodeling, and ultimately lead to worse visual outcomes.

Incorporating genetic profiling into RD management offers promising opportunities for advancing personalized therapeutic strategies. Stratifying patients according to genetic risk could facilitate targeted preventive interventions, enhanced postoperative monitoring, and specific anti-inflammatory or neuroprotective therapies. Integrating genetic information into clinical decision-making may help optimize anatomical success and long-term visual recovery [12,102,108,109,110]. However, the implementation of these strategies faces essential limitations. Currently, routine genetic screening is not standard practice in ophthalmology, and many of the proposed genetic markers lack validation across diverse populations. These gaps in clinical translation may delay the widespread adoption of personalized approaches in RD management. This personalized approach holds the potential to redefine therapeutic strategies and improve visual prognosis after RD surgery.

## 9. Inflammatory and Neurodegenerative Effects of Intraocular Tamponade in RD

While individual genetic predisposition shapes the biological response to RD and its complications, clinical management strategies must also consider extrinsic factors that can exacerbate retinal injury. Among these, the choice of intraocular tamponade agents during surgery has emerged as a crucial determinant of long-term retinal health and visual function. After PPV, the use of a tamponade agent, either gas or silicone oil, is usually necessary. The leading gases include air, sulfur hexafluoride (SF_6_), hexafluoroethane (C_2_F_6_), and octafluoropropane (C_3_F_8_) [115]. The main advantage of using gas as a tamponade agent is that it dissipates spontaneously, usually within several weeks, while silicone oil is permanent and necessitates subsequent surgery for removal [116]. Several studies have shown that using air as a tamponade yields comparable visual and anatomical results to those obtained with other gases, with the added benefit of faster postoperative recovery [117].

### 9.1. Long-Term Structural Impact of Tamponade Agents

Regarding the long-term structural impact, multiple OCT studies have evaluated the thickness of individual retinal layers in patients undergoing vitreoretinal surgery for RRD. The findings suggest that the type of buffering agent influences the structural integrity of the retina. The thickness of retinal layers is significantly preserved long-term in eyes in which gas is used compared to those in which silicone oil is used [118]. Not only has silicone been shown to impact retinal thickness reduction significantly, but decreased thickness of the ganglion cell layer (GCL), OPL, and ONL are closely related to worse visual acuity [119]. However, a study by Gharbiya et al. [120] demonstrates a decrease in macular area GCL and IPL thickness in patients undergoing vitrectomy using C_3_F_8_ compared to scleral surgery. In addition, recent research suggests that the type of tamponade also has an impact on the incidence and progression of cystoid macular edema. After phacoemulsification surgery in vitrectomized patients, cystoid macular edema tends to have a longer course and treatment if there is a history of tamponade with silicone oil compared to gas [121].

Studies in animal models have also provided relevant information on the toxicity of gases used as endotamponators, showing structural and biochemical alterations. Vieira de Souza et al. [122] studied retinal toxicity after intravitreal injection of SF_6_ and perfluorocarbon liquid (PFCL) in rabbit eyes with a balanced salt solution (BSS) control group. The SF_6_ and PFCL groups showed a significant increase in L-glutamate in the vitreous (*p* < 0.05). However, the clinical significance of this finding in humans remains uncertain. To date, there is limited direct evidence from human studies confirming neurotoxicity or retinal thinning attributable to gas tamponades such as C_3_F_8_ or SF_6_. No consistent pattern of glutamate-mediated retinal damage has been established in clinical settings. Therefore, while these experimental findings are noteworthy, their translational relevance to human patients undergoing vitrectomy with gas tamponade remains to be fully elucidated. Histological findings revealed mild changes in the SF_6_ group and significant lesions in the PFCL group, including disruption of PR outer segments, thinning of the OPL and IPL, reduced nuclei in the INL and GCL, edema, and the presence of macrophages in the superficial layers. No relevant alterations were observed in the control group [122].

Along the same lines, Doi et al. [123] evaluated histopathologic changes in the retina of rabbits after injection of C_3_F_8_ gas, SF_6_ gas, or air into the vitreous cavity. In eyes that received C_3_F_8_, thinning or disappearance of the OPL was observed in the superior retina, whereas the inferior retina remained unchanged. After injection of SF_6_ gas or air, no changes were observed in the superior or inferior retina compared to controls. Immunohistochemical examination showed an abnormal distribution of glutamate in the superior retina of eyes in which C_3_F_8_, SF_6_, or air was used. However, in the inferior retina, the distribution of glutamate was similar to that of controls in all cases. These findings suggest that intraocular gases induce histopathologic changes, especially in the superior retina, in continuous contact with the retinal surface. Noted, this retinal damage is possibly due more to mechanical damage than to the toxicity of the gas itself [123].

### 9.2. Complications of Silicone Oil Tamponade

The choice of buffering agent in vitreoretinal surgery has a significant impact on retinal structural and functional outcomes. Available evidence suggests that the use of gas, compared to silicone oil, is associated with better preservation of retinal layer thickness, a lower need for interventions, and, therefore, better long-term functional and visual outcomes. Thus, gas is presented as a buffering agent with relevant anatomical and visual recovery advantages [115,124]. However, using intraocular silicone oil remains an option as an intraocular tamponade in managing RD, especially in complex cases. However, its use is associated with various complications that may compromise long-term visual function. Several studies have documented anatomic and functional problems ranging from direct toxicity on retinal cells to silicone oil migration into adjacent structures [125].

#### 9.2.1. Inflammatory Response Mechanisms

According to Mackiewicz et al. [126], there are four main mechanisms involved in the inflammatory reaction caused by high molecular weight silicone oil: direct toxicity and immunogenicity, toxicity due to impurities or instability of the agent, emulsification of the oil, and mechanical injury due to gravity. This inflammatory response plays a crucial role in the pathogenesis of short- and long-term complications. Russo et al. [127] and Morescalci et al. [128] reported that the use of silicone oil, particularly that of heavy formulation, is associated with increased release of inflammatory mediators, exceptionally if maintained for long periods. This finding was confirmed in a prospective study by Semeraro et al. [129], which showed significantly higher levels of prostaglandin E2 and IL-1α in patients who had received heavy silicone oil as a buffer, potentially contributing to retinal toxicity.

#### 9.2.2. Retinal Damage and Visual Acuity Loss

Christensen et al. [130] documented severe visual acuity loss after silicone oil withdrawal, establishing a relationship with thinning of the inner retinal layers, especially in the macular region. This phenomenon suggests direct neuronal damage associated with silicone use. Caramoy et al. [131] conducted a study on retinal thinning in eyes with silicone oil, confirming that silicone can alter the inner retinal layers after vitrectomy; a conclusion also reached by Pichi et al. [132], especially in the macular region, which could be associated with central visual loss. In turn, Tode et al. [133] in their retrospective case series study investigated the frequency and pathophysiology involved in visual acuity loss in macula ON retinal detachments and also observed by OCT a thinning of the inner retinal layers. In the same vein, Purtskhvanidze et al. [134] and Raczyńska et al. [135] demonstrated that the impact of silicone oil is reflected by a decrease in the thickness of the ganglion cell complex, suggesting a direct toxicity on retinal neurons.

#### 9.2.3. Silicon Oil Migration as a Long-Term Neuronal Complications

In addition, Grzybowski et al. [136] reviewed long-term neuronal complications, highlighting the migration of silicone oil into the optic nerve and, in rare cases, into the central nervous system. This phenomenon can trigger irreversible optic nerve damage, especially if there are previous optic nerve abnormalities or glaucoma. Another research group [127] remarked that there are differences in the response that may be triggered depending on whether the silicone is heavy or not. In the same line, Budde et al. [137] demonstrated by histopathological analysis of enucleated eyes, the presence of silicone in the optic nerve, and that this migration was associated with granulomatous-type reactions, being able to induce inflammatory damage in attached tissues. Furthermore, Klettner et al. [138] showed that emulsified silicone oil is taken up by microglia, inducing a proinflammatory response with secretion of IL-6 and IL-8 that could aggravate retinal injury.

Taken together, these studies [125,127,128,129,130,131,132,133,134,135,136,137,138] evidence that although silicone oil is a valuable tool in managing the most complex cases of RD, its complications at the anatomical level (such as thinning of retinal layers and optic nerve degeneration), exacerbated inflammatory response, and silicone migration must be carefully considered to optimize both the choice of buffering agent and postoperative outcomes follow-up.

## 10. Current Treatments and Future Perspectives

The high inflammatory activity triggered after RRD has led to the search for adjuvant therapies that can help in the functional recovery of retinal structures. Corticosteroids have been studied as anti-inflammatory therapies in the preoperative, intraoperative, and postoperative phases of RRD surgery [139]. Preoperative corticosteroids after a choroidal detachment associated with RRD could cover the triggered inflammatory response and hypotony resulting from changes in the vasculature [140,141]. On the other hand, better functional outcomes were observed in eyes treated immediately with PPV without prior corticosteroid administration compared to a group treated with oral prednisolone (1 mg/kg body weight) for 7 days before PPV.

Intraoperative use has mainly been studied in patients with previous PVR, who are at risk of developing further postoperative complications [139]. Although intravitreal injection of triamcinolone acetonide is not recommended in patients undergoing vitrectomy associated with silicone oil endotamponade [139,142], the absence of retinotoxicity has been observed in those patients in whom a different tamponade is used [139]. On the other hand, evidence for intravitreal implant therapy with dexamethasone is currently limited [143]. Similarly, oral administration of corticosteroids has shown controversial clinical results [139]. Dehghan et al. observed no visual or anatomical improvement or reduction in postoperative complications with oral prednisolone at a 1 mg/kg dose for 10 days after surgery. In contrast, Koener et al. [144] reported that oral prednisone initially at dose of 100 mg for six days, subsequently reduced to 50 mg for five days and 12.5 mg for another four days, effectively reduced the incidence of postoperative complications such as stage B PVR. Still, there is not enough clinical evidence to support its use due to the high heterogeneity of clinical variables included in the different studies and the potential confounding this may have introduced in the analysis. The use of topical corticosteroids has not demonstrated statistically significant differences in inflammatory control compared to diclofenac sodium [145].

RPE differentiation into fibroblasts increases the risk of developing PVR and new DR. In addition, activation of glial cells, macrophages, and T lymphocytes, as well as overproduction of ECM, exacerbate fibrosis and complicate visual recovery [146]. In this context, methotrexate (MTX), an antimetabolite structural analog of folic acid with antiproliferative and anti-inflammatory properties, has been evaluated as an adjuvant treatment for PVR [147]. In that study, patients undergoing PPV for RRD received intraoperative adjuvant intravitreal infusion of MTX at a dose equivalent to 400 μg/0.1 mL. Results showed retinal repositioning rates ranging from 74% to 92%, with an average of 85% for intraoperative infusions. In addition, corrected visual acuity was improved in all MTX-treated eyes, and a significant reduction in reoperation rates for reRD (18% in MTX-treated eyes versus higher rates in those that did not receive the treatment). These findings suggest that MTX may be a promising therapeutic option for preventing PVR in patients with RRD. However, further studies are needed to confirm its efficacy and establish optimal treatment protocols.

On the other hand, different therapeutic agents have also been evaluated to palliate the inflammatory cascade that leads to this complication. Intraoperative infusion of low molecular weight heparin associated with 5-fluorouracil was tested in 174 patients [148]. The authors observed that the postoperative PVR rate in the placebo group was 26.4% and 12.6% in the combined treatment group; visual acuity was significantly better in the combined therapy group, and the number of re-interventions was higher in the placebo group. However, intraoperative complications, such as choroidal hemorrhage, were more frequent in the intervention group [148]. Another research group evaluated the antimitotic, oral colchicine 1 mg twice daily, after scleral cerclage of 184 eyes for seven weeks [149]. This randomized controlled clinical trial reported that best corrected visual acuity improved significantly in both groups with no statistically significant difference between them, as did the rate of RD secondary to PVR. In addition, Ferro Desideri et al. [150] reviewed other treatments proposed in the literature, such as antineoplastic agents (daunomycin and anti-VEGF). The evaluation of these adjuvant therapies did not show sufficient scientific evidence due to the presence of contradictory results in terms of clinical efficacy and the small and non-randomized nature of most of the studies performed.

Experimental models have shown promising results by modulating neuroinflammation and retinal neurodegeneration pathways. Insulin has been shown to increase cone survival in retinal explants [151]. Along the same lines, subretinal metformin injection in murine models also resulted in increased cone survival and decreased mononuclear phagocytes [151]. Inhibition of macrophage migration inhibitory factor (MIF) by ISO-1 has been shown to protect photoreceptors and reduce gliosis in experimental models of RD. Administration of ISO-1 resulted in a decrease in PR TUNEL-positive cells (588.68 ± 409.5 cells/mm^2^ vs. 4125.89 ± 849.5 cells/mm^2^) and a reduction in glial cell GFAP expression intensity after two weeks [152].

Moreover, exogenous administration of IL-10 (at doses of 20, 50, and 100 µM) in male Wistar rats subjected to RRD reduced RPE proliferation by a dose-dependent increase in caspase 3 production [75]. Similarly, the concentration of proinflammatory cytokines such as IL-1β, IL-6, and VEGF was regulated directly by the exogenous dose of IL-10 [75]. Expression of the microRNA miR-377 is significantly increased under conditions of inflammation and hypoxia. The miR-377 causes the activation of the NF-κB pathway, evidenced by the increase of the proteins p-IκBα, nuclear p65, and p-p65. This expression is regulated by the *SIRT1* gene that inhibits cell proliferation, cell cycle transition, migration, proinflammatory cytokine expression, and angiogenesis [153]. These authors are studying whether positive regulation of the *SIRT1* gene could inhibit the NF-κB pathway and restrict the inflammatory cascade in retinal ischemia [153].

Despite these promising experimental strategies targeting neuroinflammation and retinal degeneration, their translation into effective clinical treatments remains challenging, underscoring the urgent need to explore diverse therapeutic avenues that can be synergistically integrated to achieve a robust and sustained postoperative inflammatory control. Among the main barriers are potential toxicity and the difficulty of safe local administration (e.g., into the subretinal space), which limits the extrapolation of results obtained in animal models to human patients. This highlights the urgent need to explore diverse therapeutic strategies that can be synergistically integrated to achieve robust and sustained postoperative inflammatory control.

Given the emerging evidence linking genetic polymorphisms to the regulation of inflammatory and apoptotic responses after RD, it is plausible that individual genetic profiles could influence the efficacy of anti-inflammatory and neuroprotective therapies. Thus, future clinical trials should consider incorporating patient’s genetic stratification to assess the efficacy of novel therapeutic combinations to move towards a truly personalized treatment approach after RD. However, several challenges remain. A major hurdle is the limited availability of large-scale genomic and transcriptomic datasets specific to RD, which are essential for identifying actionable molecular targets. Additionally, the heterogeneity of RD etiology and the multifactorial nature of PVR complicate therapeutic stratification. Future efforts should also focus on developing integrative multi-omics platforms that combine genomics, proteomics, and metabolomics to better understand individual disease trajectories. Moreover, advancing the use of in vivo imaging biomarkers and AI-based predictive models may help refine patient selection and treatment timing. Finally, collaborative translational research is needed to validate these approaches in clinically relevant models before their implementation in personalized medicine protocols.

## 11. Conclusions

RD poses not only a surgical challenge but also initiates a cascade of inflammatory and neurodegenerative processes that profoundly impact visual outcomes. Accumulating evidence highlights cytokine dysregulation, oxidative stress, and glial activation, particularly involving Müller cells and microglia, as key drivers of retinal injury and impairments in recovery after RD. Additionally, persistent inflammation and the potential neurotoxic effects associated with tamponade agents, notably silicone oil, may further compromise retinal integrity and long-term function.

Although surgical repair remains the cornerstone of RD management, recent insights emphasize the need to address biological factors that influence postoperative recovery. Genetic predispositions that modulate inflammatory and apoptotic responses could provide valuable opportunities for personalized therapeutic approaches, risk stratification, and tailored follow-up strategies. Comprehensive RD care should combine surgical expertise with active modulation of molecular pathways involved in inflammation, apoptosis, and oxidative stress. However, the clinical integration of these experimental strategies remains limited at present. Future studies should also validate the clinical utility of genetic profiling and determine how it can be effectively integrated into practical treatment algorithms. Such advances will be crucial for enhancing both anatomical success rates and long-term functional outcomes for patients affected by RD.

## Data Availability

No new data were created or analyzed in this study.

## Abbreviations

The following abbreviations are used in this manuscript:

4-HNE	4-hydroxynonenal
AIF	Apoptosis-inducing factor
bFGF	Basic fibroblast growth factor
BSS	Balanced salt solution
C_2_F_6_	Hexafluoroethane
C_3_F_8_	Octafluoropropane
CTGF	Connective tissue growth factor
CXCL8	C-X-C Motif Chemokine Ligand 8
EMC	Extracellular matrix
EMT	Epithelial–mesenchymal transition
FGF	Fibroblast growth factor
Fox0	Forkhead box 0
GCL	Ganglion cell layer
GFAP	Glial fibrillary acidic protein
GSDM	Gasdermin D
GTE	Green tea extract
GWAS	Genome-wide association studies
HIF-1α	Hypoxia-inducible factor 1α
HtrA1	High temperature requirement factor A1
IL-1	Interleukin-1
IL-10	Interleukin-10
IL-6	Interleukin-6
ILs	Interleukins
INL	Inner nuclear layer
IPL	Inner plexiform layer
MCP-1	Monocyte Chemoattractant Protein
MIF	Macrophage migration inhibitory factor
MTX	Methotrexate
*MYO1F*	Myosin 1F
NOX	NADPH oxidase
ONL	Outer nuclear layer
OPL	Outer plexiform layer
OPN	Osteopontin
Par-2	Proteinase-activated receptor
PDGFR-α	Platelet-derived growth factor receptor Alpha
PFCL	Perfluorocarbon liquid
PPV	Pars plana vitrectomy
PR	Photoreceptors
PVR	Proliferative vitreoretinopathy
RD	Retinal detachment
RIP	Receptor-interacting protein kinases
ROS	Reactive oxygen species
RPE	Retinal pigment epithelium
RRD	Rhegmatogenous retinal detachment
SF_6_	Sulfur hexafluoride
TGF-β	Transforming growth factor-beta
TNF-α	Tumor necrosis factor alpha
VEGF	Vascular endothelial growth factor
